# Viburnum Opulus, a Proving

**Published:** 1881-03

**Authors:** H. C. Allen

**Affiliations:** Ann Arbor


### VIBURNUM OPULUS.—A FRAGMENTARY PROVING.

BY H. C. ALLEN, M.D., ANN ARBOR.

Viburnum Opulus.—Linn, (Vib. Oxycoccus, of some authors).

*Natural order*, Caprifoliaceæ.

*Common name*, Cranberry Tree.

Grows on low grounds, along streams; common northward and southward in the Alleghanies to the borders of Maryland, (and in Canada, Northern and Western States.) Flowers in June and July. The acid fruit is a substitute for cranberries, whence the name, *High Cranberry-bush.* The well-known *Snow-Ball Tree*, or Guelder-Rose, is a cultivated state, with the whole cyme turned into showy sterile flowers, (Gray).

The Viburnum Prunifolium, Linn, (Black Haw) which is found in dry copses from New England, Onterio and Illinois on the north, to Tennessee and Virginia on the south, is a much larger shrub and should not be confounded with V. Opulus.

*Preparation.* Tincture from the inner bark of shrub and bark of roots.

*Authorities.* (1). H. C. Allen, M.D.; (2), Miss L. F. W.; (3), Miss E. A. C.; (4), Miss E. M. S.; (5), S. E. Burchfield; (6), H. K. Brasted; (7), E. D. Bottorf, (the last five medical students in University of Michigan); (8), Dr. G. B. Palmer, N. Y. State Transactions, Vol. 15, p. 68, clinical verification.

Mind.—Confused and unable to concentrate the mind on usual mental labor;—stupid feeling as if I could not tell where I was or what to do, on awaking in the morning.

Head.—*Dull, frontal headache.*—Dull, throbbing frontal headache, extending to the eye-balls, aggravated by mental effort and relieved by moving about.—Dull, confusing frontal headache, extending to both temporal regions, as after night watching or loss of sleep, so severe as to compel to cease mental exertion,—slight headache in forenoon.—Vertigo with inclination to turn to the left.—Severe pressing pain in right supra-obital region.—A severe frontal headache with occasional vertigo set in, continuing with severity for six or seven days, almost incapacitating for study, accompanied by profuse and frequent urination.—Dull, supra-orbital and frontal headache with profuse flow of clear watery urine.—Severe pain in head just over eyes on opening them; the soreness extending back into the head.—Dull, throbbing headache continued all the evening and was so severe on retiring that I felt sick "all over".—Dull frontal headache (very uncommon with prover).

Eyes.—Sore feeling in eye-balls.—Heaviness over the eyes and in the eye-balls, so severe that at times would have to look twice to be sure of seeing an object.

Ears.—Sharp jerking pains in ears as if stabbing with a sharp knife, lasting nearly an hour.

Stomach.—Deathly sick feeling at stomach as if she could not live, every night, not relieved by any position (after leaving off the drug).—Deathly sickness at the stomach, principally at night (from repeated doses of 30 x dilution).

Abdomen.—severe sticking, darting pain in left hypochrondrium, deep seated as if in spleen, with a sensation as if some hot fluid were running through the splenic vessels, relieved by walking about the room, (evening of third day).—Intense pain in region of spleen producing faintness and relieved by perspiration.—Severe throbbing pain under floating ribs of left side, relieved by pressure, and walking about the room, (11 P.M. of third day until 3 A.M. of fourth day).—Violent throbbing in left hypochrondrium if attempted to lie on left side; could not lie on left side at all.—Whole abdomen tender and sensitive to pressure especially about umbilicus.—Rumbling, darting pains in bowels.—Cramping colic pains in lower abdomen (during mensturation).

Stool.—*Constipation.* Stool small, dry, and composed of hard, round balls, evacuated with much effort.—Great inactivity of the rectum; no inclination to stool. The constipation continued over three weeks after the drug was discontinued; no change in diet either during or after the proving.—Diarrhœa, profuse, watery, four or five stools in an hour, accompanied by chills and cold sweat.

Urinary Organs.—Profuse flow of clear watery urine all the afternoon; compelled to urinate every hour, (afternoon of third day).—Urine profuse, and pale (third night) specific gravity 1021 (third day).—Urine profuse, clear, watery and must be voided every hour or two; specific gravity 1019 (A.M. of fourth day) urine clear.—Profuse flow of watery urine (second day after 10 drops 1 x dilution).—Frequent urination of clear, watery urine all the afternoon.—One and a half hours after third dose (10 drops 1 x dilution) third day, a profuse discharge of pale, watery urine, repeated quite regularly every hour during afternoon and evening.—Fourth day, following each act of urination, was a constant sensation as if urine continued to flow.

Sexual Organs.—Male. Severe pain and swelling of epididymis and left testicle.—Right epididymis so painful and swollen (the following day) that he was obliged to use a suspensory bandage.—(This prover has been subject to attacks of epididymitis from exposure to cold or violent exercise, but in this case had not been exposed to either).—Female. Uneasy sensation in pelvic region and slight bearing down pains, continuing all the week; (from 10 drops 30 x every A.M. for a week).—Pain in back, loins, and "across me" as if menses were coming on; aggravated in early part of evening, and in a close room; ameliorated in open air and by moving about.—Was "unwell" during second week while taking the remedy; but felt so perfectly free from pain and uneasiness so peculiar to that period, that I attributed my freedom from pain to the action of the remedy.—Bearing down pains as during menstruation, with heavy, aching pain in sacral region and over pubes.—Severe bearing down pains as during menstruation, accompanied by drawing pains in anterior muscles of the thighs, and occasional sharp shooting pains over ovaries (second day after taking 20 drops (1 x) morning and evening) repeated each day after 3 P.M.—Two days after discontinuing the drug the above pains were repeated in the morning with great nervousness; could not sit or lie still but for a few minutes at a time on account of the pain.—Three days after discontinuing drug, the above mentioned pains and nervous restlessness continued, with slight flowing, (was unwell just two weeks before).—Flow lasted two days, in all respects like normal menstruation.—Was taken "unwell" a week too soon, and without usual suffering (feel badly usually day and night before).—Cramping colic pains in lower abdomen almost unbearable, coming on suddenly, with terrible severity (I never had anything like it before).—Flow ceased entirely for several hours; and then the discharge came in four large clots the color of raw beef and as solid as liver.—Cramping pains in lower abdomen as if going to be "unwell" (from repeated doses of (30 x) dilution).—"Excruciating colicky pain through the womb and lower part of the abdomen, coming on quite suddenly, just preceding the menstrual flow, sometimes lasting for ten or twelve hours, relieved by the 1 x dilution."—"Pains beginning in the back and going around to the loins (?) and across to the pubic bone, like labor pains have been relieved promptly."

CHEST.—Sharp shooting pain in left chest over 6th rib near the sternum.—During the proving, an old heart trouble, an irregularity of the pulse—remission of third beat—of which I had felt nothing for over six months, returned.—Felt as if her breath would leave her body, and her heart would cease beating (during menstrual colic).

NECK AND BACK.—Tired bruised pain in muscles of the back, extending from point of scapula to wing of ilium on each side of the spine, relieved by leaning against chair or firm pressure.—Wandering, tired pains extending to hips and knees with disinclination to move or walk about.—Severe pain in the back (region of the spleen) rendering ordinary work (in the labratory) difficult; relieved by pressing across the back with arms crossed.—Pain in back repeated next day less severely (without repeating the dose).

HANDS.—Strange buzzing feeling in hands as if they would burst.

SLEEP.—Restless and unrefreshing sleep.

GENERALITIES.—Could not lie on affected side.—The muscles of entire left side of body sore as if bruised or strained by over lifting.—Muscles of back lame and bruised as after severe physical exertion. Inability to lie on the left side during entire proving. Tired in the morning on rising.

